# Radiation Therapy for Relapsed or Refractory Diffuse Large B-Cell Lymphoma: What Is the Right Regimen for Palliation?

**DOI:** 10.1016/j.adro.2022.101016

**Published:** 2022-07-03

**Authors:** Christopher M. Wright, Alexandra D. Dreyfuss, Jonathan A. Baron, Russell Maxwell, Amberly Mendes, Andrew R. Barsky, Abigail Doucette, Jakub Svoboda, Elise A. Chong, Joshua A. Jones, Amit Maity, John P. Plastaras, Ima Paydar

**Affiliations:** a Department of Radiation Oncology, University of Pennsylvania, Philadelphia, Pennsylvania; b Department of Radiation Oncology, Memorial Sloan Kettering Cancer Center, New York, New York; c Department of Medicine, Hematology/Oncology Division, University of Pennsylvania, Philadelphia, Pennsylvania

## Abstract

**Purpose:**

To report objective response rates (ORR), time to local failure (TTLF), and overall survival (OS) among patients with relapsed or refractory diffuse large B-cell lymphoma after salvage- or palliative-intent radiation therapy (RT) and to investigate whether outcomes differed with conventional versus hypofractionated (≥2.5 Gy/fraction) RT.

**Methods and Materials:**

A single-institution observational cohort study was performed for patients who completed a course of RT for relapsed or refractory diffuse large B-cell lymphoma between January 1, 2008, and April 1, 2020. Predictors of ORR, TTLF, and OS were calculated using univariable and multivariable regression models. The Kaplan-Meier method was used to estimate TTLF and OS, and log-rank analysis was used to compare outcomes. Equivalent dose in 2 Gy fractions (EQD2) was calculated using an α/β of 10.

**Results:**

One-hundred and sixty-nine patients were treated with 205 RT courses (73 [36%] salvage, 132 [64%] palliative), and hypofractionated RT was used in 100 RT courses (49%). Median RT dose was 30 Gy (range, 8-60 Gy). ORR was 60% for the total cohort (53% and 69% for palliative and salvage cohorts, respectively). Over a median follow-up time of 4 months, median OS in all patients was 5 months (3 and 22 months for palliative and salvage cohorts, respectively). No statistically significant differences in ORR, TTLF, and OS were observed with hypofractionation compared with conventional fractionation. EQD2 ≥35 Gy was associated with improved ORR (odds ratio, 3.79 [1.19-12.03]; *P* = .024) and prolonged TTLF (0.39 [0.18-0.87]; *P* = .022), while double-hit receptor status (8.18 [1.08-62.05]; *P* = .042), cell of origin (3.87 [1.17-8.74]; *P* = .0012), and bulky disease (≥7.5 cm; 2.12 [1.18-3.81]; *P* = .012) were associated with inferior TTLF. In the palliative-only cohort, a low-dose regimen of 8 Gy in 2 fractions was associated with similar ORR compared with other fractionation schema but trended towards inferior TTLF (*P* = .36).

**Conclusions:**

Hypofractionation is not associated with differences in disease outcomes for patients with relapsed or refractory diffuse large B-cell lymphoma, while higher RT dose (EQD2 ≥35 Gy) may improve ORR and TTLF. Future work is warranted to elucidate the ideal dose and fractionation schema for such patients who will likely also undergo novel systemic agents and cellular therapies.

## Introduction

Diffuse large B-cell lymphoma (DLBCL) is the most common subtype of non-Hodgkin lymphoma, representing one-third of the more than 77,000 cases diagnosed annually in the United States.^1^, ^2^, ^3^ Per National Comprehensive Cancer Network (NCCN) guidelines, first-line therapy often consists of combination chemoimmunotherapy followed by consolidative involved site radiation therapy (RT) in select cases.^4^ After initial therapies, approximately 10% to 15% of patients exhibit primary refractory disease, and an additional 20% to 25% subsequently relapse.^5^ Relapsed or refractory diffuse large B-cell lymphoma (r/rDLBCL) may be salvaged with second-line chemotherapy followed by autologous stem-cell transplant; however, outcomes are relatively unfavorable, with a progression free survival of less than 50% within 3 years posttransplantation.^6^, ^7^, ^8^, ^9^, ^10^ For disease that is refractory to or relapsed after 2 lines of systemic therapy, anticluster of differentiation 19-targeted chimeric antigen receptor T-cell (CART) therapy has emerged as a promising novel treatment for r/rDLBCL.^11^^,^^12^

In select cases of localized r/rDLBCL, salvage RT may be used in a curative role. Response rates to RT may exceed 80%, and durable local control has been achieved in up to two-thirds of patients at 5 years.^13^ For patients with more advanced-stage disease, RT may be used for palliation of symptomatic tumors or to provide durable control of lesions at high risk for lymphoma-related complications, such as pain, spinal cord compression, bowel or biliary obstruction, or gastrointestinal bleeding. Guidelines from the International Lymphoma Radiation Oncology Group (ILROG) provide a framework for the myriad of approaches to using RT for r/rDLBCL.^14^ For patients treated with palliative-intent RT, the ILROG guidelines suggest a hypofractionated schedule of 8 to 30 Gy depending on the resultant dose to organs at risk, tumor size, and patient performance status. Such patients have limited prognoses, with studies reporting a median survival of only several months.^13^ Thus, hypofractionated RT to symptomatic disease sites may be of particular benefit. However, data on outcomes comparing conventional versus hypofractionated RT are sparse.

Here, we report a single institution's experience of patients with r/rDLBCL treated with RT in the salvage or palliative setting. We hypothesized that hypofractionated RT is a reasonable and safe option for patients with poor prognoses and confers objective response rates (ORR) and times to local failure (TTLF) comparable to conventionally fractionated regimens. This study is the first to report outcomes after salvage or palliative courses of NCCN- and ILROG-endorsed hypofractionated RT regimens compared with protracted, conventionally fractionated RT regimens for r/rDLBCL.^4^^,^^14^

## Methods and Materials

We performed a single-institution observational cohort study of patients ≥18 years old treated with RT in the salvage or palliative setting for DLBCL between January 1, 2008, and April 1, 2020 at the Univeristy of Pennsylvania. Patient courses of RT were classified as salvage or palliative per the discretion of the treating radiation oncologist as described in the patient's electronic medical record. Salvage RT was generally defined as either (1) treatment for locoregionally confined gross disease with curative or definitive intent or (2) as a bridging therapy to subsequent systemic therapeutics. Palliative RT courses were delivered for symptom relief or to prevent future oncologic emergencies (eg, neurologic deficit, bowel obstruction, or hemoptysis). Patients without a pathologically confirmed diagnosis of DLBCL, patients who received central nervous system or skin-directed RT (given differing natural histories), and patients who were treated with consolidative RT were excluded. This study was approved by the University of Pennsylvania institutional review board.

Patient-specific variables were collected and recorded including age, sex, race, initial stage at diagnosis, and Eastern Cooperative Oncology Group (ECOG) performance status at the time of RT. Disease-specific variables were collected and recorded including activated B-cell (ABC) or germinal center B-cell (GCB) cell of origin; gene rearrangements of MYC, BCL2, and BCL6 by fluorescence in situ hybridization; overexpression of the MYC, BCL2, and/or BCL6 by immunohistochemistry; and largest dimension of the treated mass. Bulky lesions were defined as a maximal diameter of ≥7.5cm. Treatment-related variables collected and recorded included treatment intent, year of RT, treating department (main site or satellite), site of RT, dose delivered, fractionation, and the number of lines of prior systemic therapies. With respect to dose delivered, equivalent dose in 2 Gy fractions (EQD2) was calculated using an α/β ratio of 10, and RT courses were analyzed by the following groupings: EQD2 <20 Gy (low), 20 to 35 Gy (moderate), and ≥35 Gy (high). The commonly used fractionation scheme of 8 Gy in 2 fractions (EQD2 = 9.3 Gy) was also compared against other RT fractionations.

Outcomes of interest included ORR, defined as achieving either a complete response (CR) or partial response on post-RT computed tomography or positron emission tomography/computed tomography. In-field treatment responses to the treated lesion(s) were classified per the Lugano criteria.^15^ Other outcomes included TTLF and overall survival (OS). Follow-up was calculated as the number of months from the end of the RT treatment course to the last documented follow-up in the electronic medical record or death. For patients with multiple courses of RT, ORR was captured for each lesion independently. TTLF, using course-level data, was defined as time in months from RT end date to local progression of the irradiated index lesion with censoring at date of last follow-up or death. OS, using patient-level data, was defined as time in months from end date of the initial RT course to death with censoring at last follow-up date.

Patients were divided into 2 groups: conventionally fractionated RT (fraction sizes <2.5 Gy) and hypofractionated RT (fraction sizes ≥2.5 Gy). Disease outcomes were assessed for all patients with respect to fractionation schemes (conventional vs hypofractionated). RT toxicity was also analyzed and prospectively recorded and graded per the Common Terminology Criteria for Adverse Events version 5.

### Statistical analysis

All statistical analyzes were carried out using SAS software, version 9.4 (SAS Institute, Cary, NC), while figures were constructed using GraphPad Prism 9.3 (GraphPad Software Inc, San Diego, CA). Descriptive statistics were used to assess continuous (median values, ranges, and interquartile ranges) and categorical (frequency counts and proportions) variables. Continuous and categorical variables were compared among subgroups using the nonparametric Mann-Whitney *U* and Fisher's exact tests, respectively. Univariable (UVA) and multivariable (MVA) logistic regression models were used to assess potential associates of the odds ratio (OR) binary variable, while Kaplan-Meier curves and Cox proportional hazard regression models (UVA and MVA) were used to assess any time-to-event outcomes such as TTLF and OS. Log rank analysis was used to compare outcomes for TTLF and OS. Only variables meeting a predetermined *P* < .10 cut-off on UVA were considered candidates for the final MVA models. Patients with missing variable data were excluded from any univariable analyses (eg, Fisher's exact and Mann-Whitney *U* tests) but were included in MVA models to preserve power. Statistical tests were considered significant if associated 2-tailed *P* values were less than a predetermined type I error rate set at .05.

## Results

### Patient, disease, and treatment characteristics

**Figure 1:**
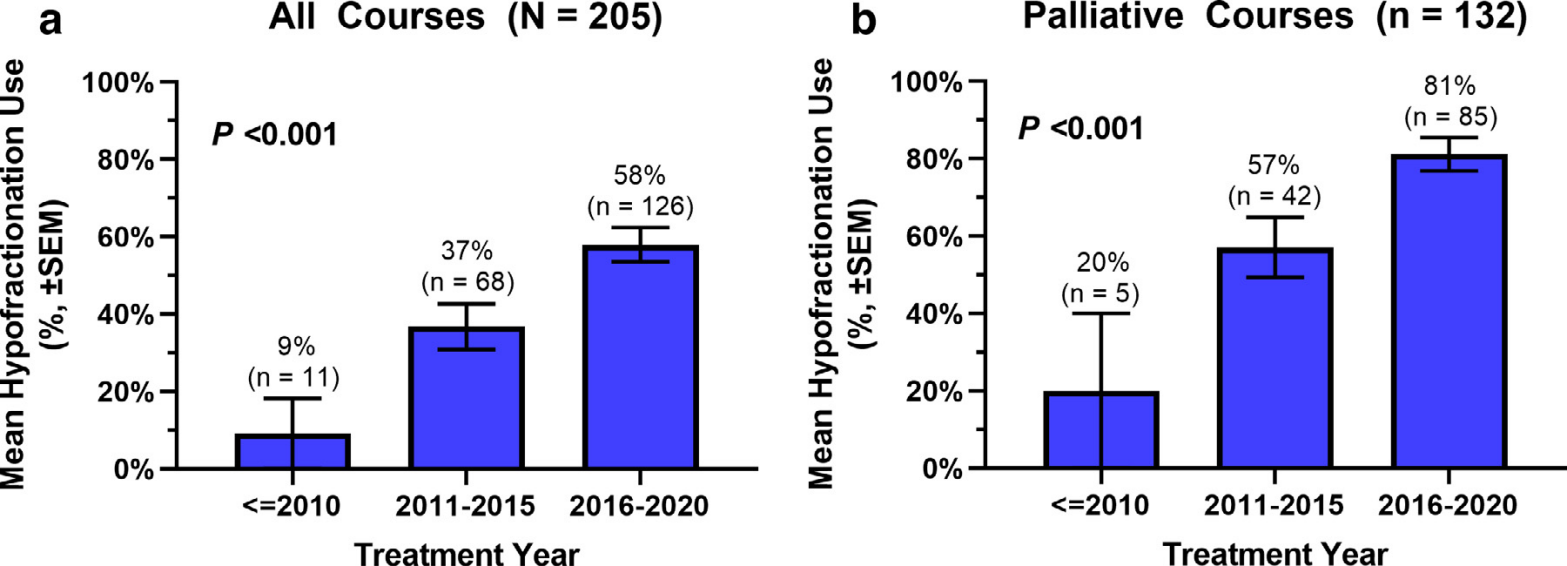
Use of hypofractionated radiation therapy by treatment year for all courses and palliative courses.

A total of 169 patients (50% >65 years) and 205 RT courses were included in this analysis (Table 1), of which 106 (52%) were delivered with conventional fractionation and 99 (48%) were delivered with hypofractionated RT. Of the total number of RT courses included, 132 (64%) were delivered with palliative intent. Factors associated with hypofractionated RT included patients who received treatment during or after 2016 (Fig. 1, *P* < .0006) and palliative intent RT (*P* < .0001), among others (Table 1). The most commonly used regimens were 39.6 Gy (1.8 Gy x 22 fractions) and 20 Gy (4 Gy x 5 fractions) for the conventionally fractionated and hypofractionated cohorts, respectively (Fig. E1).

**Table 1:** Baseline patient, disease, and RT treatment characteristics for all courses and stratified by fractionation

Characteristics* RT course-level data of 169 unique patients n (%)	All courses (n = 205%)	HFX (≥2.5 Gy/fx) (n = 99, 48%)	CFX (<2.5 Gy/fx) (n = 106, 52%)	*P*
**Patient details**				
Age at RT				
<65 y	102 (50)	49 (49)	53 (50)	1.00
>65 y	103 (50)	50 (51)	53 (50)	
Sex				
Male	113 (55)	56 (57)	57 (54)	.78
Female	92 (45)	43 (43)	49 (46)	
Race				
White	176 (86)	88 (89)	88 (83)	.52
Black or African American	15 (7)	6 (6)	9 (9)	
Other	14 (7)	5 (5)	9 (8)	
ECOG PS				
0-2	159 (78)	67 (68)	92 (87)	.0002
3-4	37 (18)	28 (28)	9 (8)	
Missing	9 (4)	4 (4)	5 (5)	
**Disease details**				
Double hit				
Yes	32 (16)	16 (16)	16 (15)	.83
No	69 (34)	37 (37)	32 (30)	
Missing	104 (51)	46 (46)	58 (55)	
Double expressor				
Yes	75 (37)	44 (44)	31 (29)	.31
No	66 (32)	33 (33)	33 (31)	
Missing	64 (31)	22 (22)	42 (40)	
Cell of origin				
ABC	70 (34)	39 (39)	31 (29)	.45
GCB	114 (56)	56 (57)	58 (55)	
Missing	21 (10)	4 (4)	17 (16)	
Bulky (>7.5 cm)				
Yes	74 (36)	34 (34)	40 (38)	1.00
No	109 (53)	50 (51)	59 (56)	
Missing	22 (11)	15 (15)	7 (7)	
Systemic treatment lines				
≤2	115 (56)	48 (48)	67 (63)	.036
>2	90 (44)	51 (52)	39 (37)	
**RT treatment details**				
Intent				
Salvage	73 (36)	5 (5)	68 (64)	< .0001
Palliative	132 (64)	94 (95)	38 (36)	
Dose				
<20 Gy	45 (22)	32 (32)	13 (12)	< .0001
20-35 Gy	72 (35)	49(49)	23 (22)	
>35 Gy	88 (43)	18 (18)	70 (66)	
EQD2				
<20 Gy	44 (21)	31 (31)	13 (12)	< .0001
20-35 Gy	73 (36)	50 (51)	23 (22)	
≥35 Gy	88 (43)	18 (18)	70 (66)	
RT site				
Head & neck	26 (13)	10 (10)	16 (15)	.0007
Thorax	33 (16)	14 (14)	19 (18)	
Abdomen/pelvis	74 (36)	25 (25)	49 (46)	
Spine	29 (14)	19 (19)	10 (9)	
Extremities	23 (11)	18 (18)	5 (5)	
Multiple sites	13 (6)	9 (9)	4 (4)	
Other	6 (3)	4 (4)	2 (2)	
Missing	1 (<1)	0 (0)	1 (1)	
RT technique				
Electron	7 (3)	6 (6)	1 (1)	< .0001
3D-CRT	112 (55)	72 (72)	40 (38)	
IMRT/VMAT	57 (28)	14 (14)	43 (41)	
Proton	14 (7)	2 (2)	12 (11)	
Combined	11 (5)†	3 (3)	8 (8)	
Missing	4 (2)	2 (2)	2 (2)	
RT completion				
Yes	172 (84)	84 (85)	88 (83)	.85
No	33 (16)	15 (15)	18 (17)	
Treatment location	160 (78)	80 (81)	80 (75)	.40
Main site	160 (78)	80 (81)	80 (75)	.40
Satellite	45 (22)	19 (19)	26 (25)	
Treatment year				
≤2015	79 (39)	26 (26)	53 (50)	.0006
>2015	126 (61)	73 (74)	53 (50)	
Follow-up time (months)	All courses	Palliative courses	Salvage courses	
Median	4	3	11	
(Range, IQR)	(0-118, 1-15)	(0-96, 1-9)	(0-118, 3-23)	
Missing	12/205 (6)	4/132 (3)	8/73 (11)	

*Abbreviations:* 3D-CRT = 3D conformal radiation therapy; ABC = activated B-cell; CFX = conventional fractionation; ECOG = Eastern Cooperative Oncology Group; EQD2 = equivalent dose in 2 Gy fractions; fx = fraction; GCB = germinal center B-cell; HFX = hypofractionation; IMRT = intensity modulated radiation therapy; IQR = interquartile range; PS = performance status; RT = radiation therapy; VMAT = volumetric modulated arc therapy.

⁎ Missing values included for completeness and viewing but excluded from statistical analysis. Some percentages may not exactly add to 100% with rounding.

† Combination RT technique further details (n = 11): 3D-CRT-electron (n = 1), 3D-CRT-IMRT/VMAT (n = 6), proton-3D-CRT (n = 1), and proton-IMRT/VMAT (n = 3).

### Disease-control outcomes and predictors of ORR and TTLF

Median OS for the total cohort was 5 months after a median follow-up of 4 months (interquartile ranges, 1-16 months). Median OS for the palliative and salvage cohorts was 3 and 22 months, respectively (Fig. E2; *P* < .001). The ORR for the cohort was 60% (CR in 35%), with significant differences in ORRs for bulky disease and palliative- versus salvage-intent courses of RT (Table 2).

**Table 2:** Treatment response in all courses and stratified by fractionation, intent, and bulky tumor size (≥7.5 cm)

		Fractionation		Intent		Tumor size	
Response, n	All courses* (n = 159)	CFX (n = 88)	HFX (n = 71)	Salvage (n = 65)	Palliative (n = 94)	Nonbulky†‡ (n = 88)	Bulky†‡ (n = 58)
CR (%)	55 (35)	33 (38)	22 (31)	29 (45)	26 (28)	37 (42)	12 (21)
PR (%)	40 (25)	25 (27)	16 (23)	16 (25)	24 (26)	20 (23)	17 (29)
SD (%)	29 (18)	13 (15)	16 (23)	6 (9)	23 (25)	12 (14)	16 (28)
PD (%)	35 (22)	18 (20)	17 (24)	14 (22)	21 (22)	19 (22)	13 (22)
ORR (CR + PR) (%)	95 (60)	58 (66)	38 (54)	45 (69)	50 (53)	57 (65)	29 (50)
*P*		.51		.042		.029	

*Abbreviations:* CFX = conventional fractionation (<2.5 Gy/fraction); CR = complete response; HFX = hypofractionation (≥2.5 Gy/fraction); ORR = objective response rate; PD = progressive disease; PR = partial response; SD = stable disease.

⁎ One hundred fifty-nine of total 205 courses (78%) had available response data to analyze.

† Thirteen of included 159 courses (8%) had missing bulky data.

‡ Bulky defined as maximal diamter of ≥7.5cm.

Table 3 lists the results from the UVA and MVA of ORR and TTLF for all courses. RT fractionation (hypofractionation vs conventional fractionation) was not associated with either outcome. ECOG performance status (PS) ≤ 2, salvage treatment intent, and completion of RT course were significantly associated with improved ORR on UVA, but only EQD2 ≥35 Gy (OR, 3.79 [1.19-12.03]; *P* = .024) was associated with a higher ORR on MVA. Factors associated with inferior TTLF on MVA included double hit receptor status (OR, 8.18 [1.08-62.05]; *P* = .042), GCB cell of origin (OR, 3.87 [1.17-8.74]; *P* = .0012), bulky disease (OR, 2.12 [1.18-3.81]; *P* = .012), and thoracic site of disease (OR, 6.43 [1.69-24.46]; *P* = .0063). EQD2 ≥35 Gy (OR, 0.39 [0.18-0.87]; *P* = .022) was associated with prolonged TTLF. ECOG PS ≥3 (OR, 2.27 [1.26-4.09]; *P* = .0065), bulky disease (OR, 1.63 [1.05-2.55, 0.031]), and early termination of RT course (OR, 3.74 [2.04-6.84]; *P* < .0001) were associated with worse OS on MVA (Table E1). Hypofractionated RT regimens were associated with inferior OS on UVA but not on MVA (OR, 1.35 [0.78-2.32]; *P* = .29).

**Table 3:** UVA and MVA regression models assessing predictors of objective response and TTLF in all courses

Variable	Objective response		TTLF	
OR HR (95% CI, *P*)*	UVA	MVA	UVA	MVA
Age at RT				
≤65 y	Reference		Reference	
>65 years	0.95; (0.69-1.30, 0.74)		1.18; (0.70-1.97, 0.54)	
Sex				
Female	Reference		Reference	
Male	0.89; (0.65-1.23, 0.48)		0.92; (0.55-1.54, 0.76)	
Race				
White	Reference		Reference	
Black or African American	0.67; (0.20-2.17, 0.50)		0.70; (0.22-2.25, 0.35)	
Other	1.67; (0.31-8.89, 0.55)		0.57; (0.14-2.33, 0.62)	
ECOG PS				
0-2	Reference	Reference	Reference	
3-4	0.30; (0.11-0.80, 0.016)	0.34; (0.10-1.10, 0.072)	1.82; (0.85-3.88, 0.12)	
Double hit				
No	Reference		Reference	Reference
Yes	0.36; (0.27-1.94, 0.52)		2.26; (1.02-5.00, 0.044)	8.18; (1.08-62.05, 0.042)
Double expressor				
No	Reference		Reference	Reference
Yes	0.54; (0.25-1.17, 0.12)		1.72; (0.93-3.15, 0.082)	0.33; (0.05-2.16, 0.25)
Cell of origin				
ABC	Reference		Reference	Reference
GCB	0.57; (0.28-1.6, 0.12)		1.76; (0.97-3.17, 0.061)	3.87; (1.17-8.74, 0.0012)
Bulky (≥7.5 cm)				
No	Reference	Reference	Reference	Reference
Yes	0.54; (0.28-1.07, 0.077)	0.51; (0.24-1.07, 0.077)	2.02; (1.18-3.47, 0.010)	2.12; (1.18-3.81, 0.012)
Systemic treatment lines				
<2	Reference		Reference	
>2	0.38; (0.61-2.19, 0.65)		0.89; (0.53-1.50, 0.67)	
Intent				
Salvage	Reference	Reference	Reference	
Palliative	0.51; (0.26-0.98, 0.044)	1.21; (0.48-3.01, 0.69)	0.97; (0.58-1.63, 0.91)	
Fractionation				
CFX	Reference		Reference	
HFX	0.63; (0.33-1.19, 0.15)		1.06; (0.62-1.80, 0.84)	
EQD2				
<20 Gy	Reference	Reference	Reference	Reference
20-35 Gy	1.52; (0.62-3.72, 0.36)	1.77; (0.65-4.81, 0.26)	0.59; (0.29-1.20, 0.14)	0.51; (0.21-1.22, 0.13)
≥35 Gy	4.09; (1.71-9.78, 0.0015)	3.79; (1.19-12.03, 0.024)	0.50; (0.27-0.93, 0.029)	0.39; (0.18-0.87, 0.022)
RT site				
Head & neck	Reference		Reference	Reference
Thorax	0.39; (0.11-1.39, 0.15)		4.43; (1.27-15.43, 0.020)	6.43; (1.69-24.46, 0.0063)
Abdomen/pelvis	0.45; (0.14-1.39, 0.17)		3.35; (1.00-11.14, 0.049)	2.29; (0.63-8.29, 0.21)
Spine	0.61; (0.15-2.53, 0.50)		2.05; (0.49-8.56, 0.33)	1.40; (0.30-6.43, 0.67)
Extremities	0.42; (0.11-1.65, 0.21)		2.85; (0.73-11.04, 0.13)	2.31; (0.54-9.85, 0.26)
Multiple sites	0.50; (0.10-2.53, 0.40)		2.51; (0.56-11.22, 0.23)	2.76; (0.57-13.29, 0.20)
Other	0.67; (0.092-4.81, 0.69)		1.99; (0.21-19.29, 0.55)	2.47; (0.24-25.13, 0.44)
RT technique				
3D-CRT	Reference		Reference	
Electron	0.90; (0.17-4.75, 0.90)		1.03; (0.31-3.39, 0.97)	
IMRT/VMAT	1.75; (0.84-3.65, 0.13)		0.98; (0.54-1.80, 0.96)	
Proton	2.71; (0.68-10.76, 0.16)		1.39; (0.57-3.39, 0.47)	
Combined	1.58; (0.43-5.83, 0.49)		1.54; (0.59-4.02, 0.38)	
RT completion				
Yes	Reference	Reference	Reference	Reference
No	0.29; (0.10-0.82, 0.020)	0.50; (0.16-1.60, 0.24)	2.43; (1.18-4.99, 0.016)	1.49; (0.64-3.46, 0.35)
Treatment location				
Satellite	Reference	Reference	Reference	
Main site	0.45; (0.19-1.08, 0.072)	0.50; (0.19-1.32, 0.16)	1.18; (0.61-2.28, 0.62)	
Treatment year				
≤2015	Reference		Reference	
>2015	0.67; (0.35-1.30, 0.24)		1.22; (0.71-2.08, 0.48)	

*Abbreviations:* 3D-CRT = 3D conformal radiation therapy; ABC = activated B-cell; CFX = conventional fractionation (<2.5 Gy/fraction); CI = confidence interval; ECOG = Eastern Cooperative Oncology Group; EQD2 = equivalent dose in 2 Gy fractions; GCB = germinal center B-cell; HFX = hypofractionation (≥2.5 Gy/fraction); HR = hazard ratio; IMRT = intensity modulated radiation therapy; MVA = multivariable analysis; OR = odds ratio; PS = performance status; RT = radiation therapy; TTLF = time to local failure; UVA = univariable analysis; VMAT = volumetric modulated arc therapy.

⁎ Logistic regression models were used to evaluate predictors of objective response (159 observations, 78% of total 205 courses) with calculated ORs, while Cox proportional hazards models were used for TTLF (155 observations, 76% of total 205 courses) with calculated HRs. Patients with missing values at various covariates were included in models to maintain statistical power, but associated ORs and HRs for missing values are not shown because they were not clinically meaningful. *Italicized* variables in UVA were included in MVA as they met predetermined cut-off *P* < .10.

### Disease-control outcomes after 8 Gy in 2 fractions

**Figure 2:**
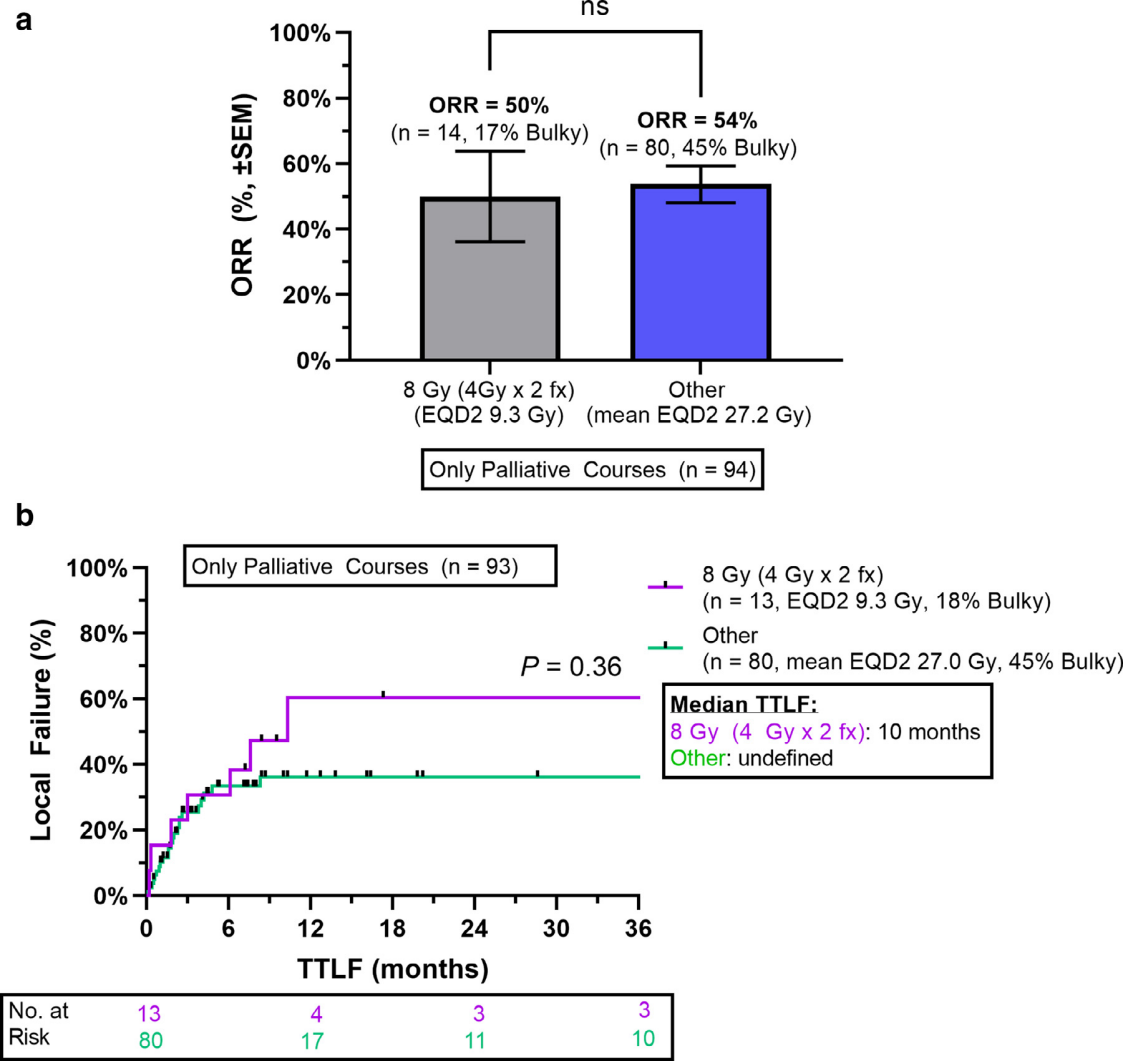
A, Objective response rates and B, time to local failure for 8 Gy in 2 fractions versus other palliative radiation therapy regimens.

Among patients treated with palliative intent, receipt of 8 Gy in 2 fractions (n = 14, EQD2 = 9.3 Gy) was not associated with ORR (50% vs 54%; *P* = 1.0) compared with all other palliative RT courses (n = 80, mean EQD2 27.2 Gy). This regimen was less frequently used for bulky lesions (17% vs 45%; *P* = .11). Median TTLF for this regimen was 10 months versus not reached for other palliative RT courses (Fig. 2; *P* = .36). Median OS was 8 and 3 months for patients receiving 8 Gy in 2 fractions versus other palliative RT regimens, respectively (*P* = .21).

### Treatment toxicity

RT was well tolerated overall, with 14 patients (7%) experiencing grade 3 toxicity and no observed grade 4 or 5 toxicities (Fig. E3). After stratifying by RT dose (EQD2 ≤ 30 Gy vs > 30 Gy), there were no significant differences in acute toxicity between the conventionally fractionated and hypofractionated groups (Fig. E3).

## Discussion

Although treatment approaches for patients with r/rDLBCL have traditionally been limited, offering long-term remission or cure in only a minority of patients, new systemic therapies continue to emerge, and modern clinical management can involve a variety of treatment strategies depending on patient, disease, and previous treatment response characteristics. We report the first large comparison of hypofractionated versus conventionally fractionated regimens for patients with r/rDLBCL receiving palliative or salvage RT. Our findings of comparable response rates and local control further support the ILROG and NCCN recommendations for use of hypofractionated RT in the palliative setting. Given the increasingly diverse and evolving role RT can play in patients with r/rDLBCL, it is paramount to optimize the efficacy and feasibility of RT strategies in nuanced clinical scenarios and to identify subgroups of patients who may benefit from either short-course or protracted RT regimens.

In this large retrospective analysis, we reported our experience treating 169 patients with r/rDLBCL (205 RT courses) with RT in the palliative or salvage setting. Coinciding with the publication of ILROG guidelines,^16^ patients were generally treated with involved site RT after 2014. We observed an objective response in 60% of patients (53% and 69% for patients treated with palliative- and salvage-intent RT, respectively). Median OS was poor at only 5 and 3 months for the total cohort and palliative cohort, respectively, compared with 22 months for the salvage RT cohort. Prior studies have reported similar results after palliative RT, albeit with somewhat more favorable response rates and survival. For example, several small, single-institutional studies have retrospectively reported 2-year local control rates of 54% to 73% and response rates as high as >80%.^13^^,^^17^, ^18^, ^19^ The decrease in response and response durability we observed in our study may in part be due to our exclusion of patients treated with RT in the consolidative setting, the fact that 44% of patients in our cohort received at least 3 lines of systemic therapy before RT, and the high percentage of palliative RT courses included in our analysis. Nevertheless, taken together with previously reported data, our findings highlight a need for effective RT regimens that can be delivered in a practical and convenient timeframe.

In the case of palliative-intent RT, ILROG and NCCN guidelines recommend hypofractionated RT courses as an ideal approach to symptomatic or quality-of-life reducing disease. Hypofractionated RT is particularly suitable for patients with troublesome or symptomatic lesions as well as when patients are in between systemic therapies, as these patients are often not well enough to undergo long courses of RT or may not have a 4- to 6-week time window for prolonged courses of RT in between systemic therapies. To date, however, strong evidence-based data supporting this recommendation are lacking. A recent population-based study of palliative RT from British Columbia (BC) analyzed 217 patients treated primarily with hypofractionated RT (81% of courses with fraction sizes ≥2.5 Gy) and demonstrated a clinical and/or radiographic response rate of 83% and a 6-month local control rate of 66.7%.^20^ Patients who did not require concurrent steroids achieved a greater response (OR, 3.5; *P* = .011), while responses to first-line systemic therapy and smaller lesion size were associated with improved local progression rates (hazard ratio, 0.2; *P* < .001 and hazard ratio, 0.5; *P* = .02, respectively). Although this series fills a substantial void in the literature, our study differs from and contributes to the available literature for several reasons: (1) here we compare disease outcomes for patients treated with hypofractionated versus conventionally fractionated regimens; (2) radiologic responses were available after 12% of the RT courses in the BC series versus 78% in our series; (3) our study represents a more heavily pretreated patient cohort (66% vs 41% of patients with ≥2 lines of systemic therapy); and (4) our cohort excluded patients treated to cutaneous lesions, which comprised 22% of the BC cohort. The higher response rates observed in the BC series (83%) may in part be driven by the improved responses observed in cutaneous lesions (OR, 6.9 on univariable analysis; *P* = .002) and the inclusion of patient symptom improvement in quantifying response. Of the 44 courses (12%) from the BC series with an available radiographic response, the 61% response rate (CR 18%) is comparable to that reported in our series (60% ORR and 35% CR). These results support the use for hypofractionated RT in the palliative setting, particularly for patients with limited life expectancies.

To the best of our knowledge, our study is among the first to report on outcomes comparing hypofractionated and protracted RT for r/rDLBCL. Here, we did not observe any significant differences on MVA for disease outcomes (ORR, TTLF, or OS) with hypofractionated RT. Hypofractionated RT was associated with inferior ORR and OS on UVA (Table E1), but not when controlling for other factors on MVA, potentially reflecting selection bias as clinicians may opt for shorter courses of RT for patients with limited life expectancies. Still, these findings lend support to the continued use of hypofractionated RT in select patients. Although the only factors we identified as significantly associated with increased use of hypofractionated regimens were ECOG PS, RT intent (palliative vs salvage), and RT administration post-2015, the latter finding is encouraging; as the use of hypofractionation increases in the coming years, identification of specific patient, disease, or treatment characteristics that benefit most from hypofractionated RT will be possible.

A secondary objective of this study was to evaluate whether disease outcomes differed according to certain cohort characteristics. Response rates differed for bulky versus nonbulky disease, with CR more likely for patients treated with RT for nonbulky disease. Interestingly, several disease characteristics were inversely associated with TTLF, including double-hit receptor status, GCB cell of origin, bulky disease, and thoracic disease. Collectively, some of these results are consistent with those published in the literature. For instance, a study of 25 patients with r/rDLBCL treated with palliative-intent low-dose RT (4 Gy in 2 fractions) found that bulky disease (>5 cm) was associated with inferior responses to RT.^21^ In contrast to the findings from our study, GCB cell of origin was associated with improved response rates. Whether higher palliative doses may be necessary for bulky disease or lymphomas with particular immunophenotypic or genetic features remains to be characterized.^22^, ^23^, ^24^, ^25^ A larger multi-institutional review is currently underway to further investigate the association between gene rearrangements or overexpression and radiosensitivity.

**Figure 3:**
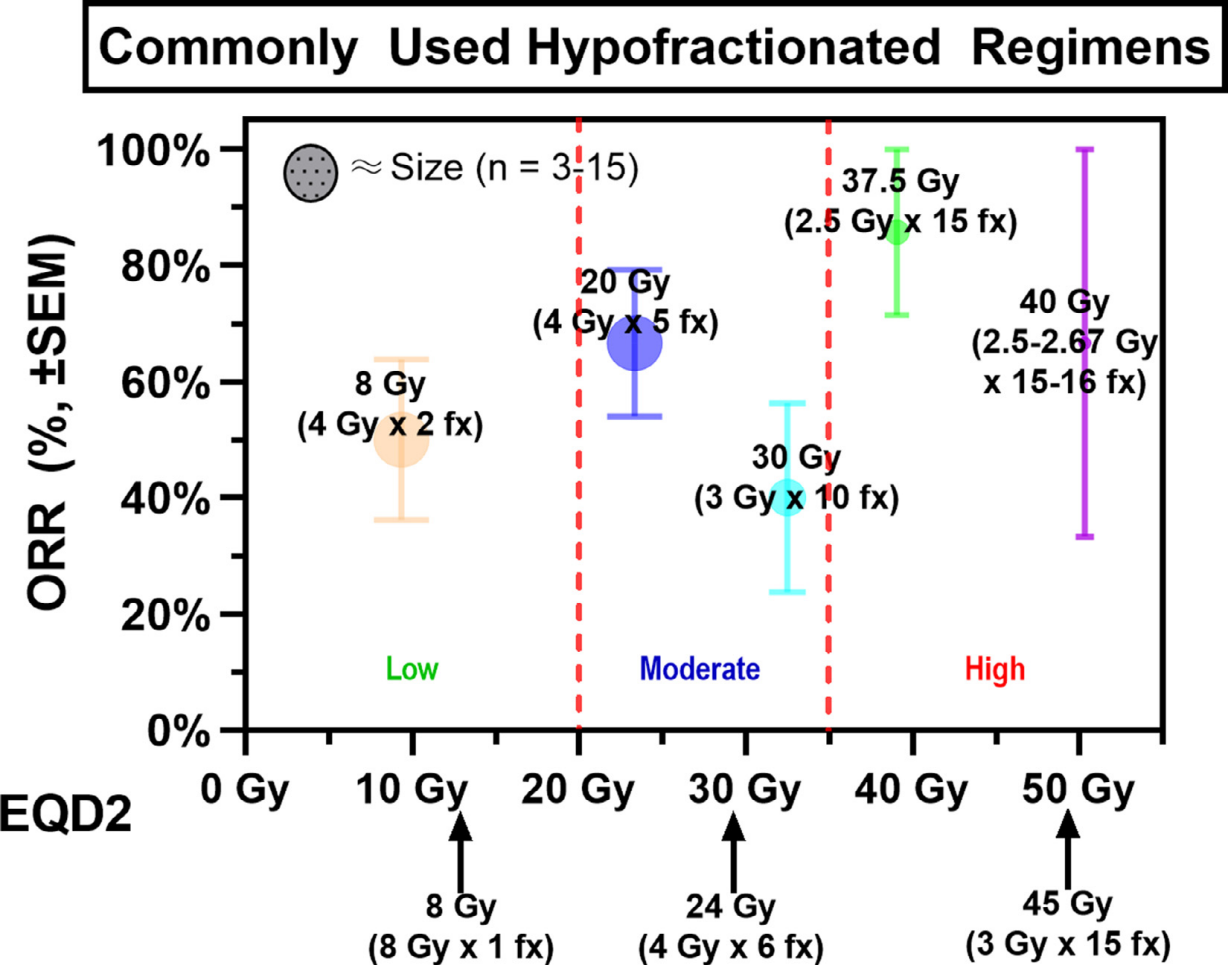
Plot of objective response rates by equivalent dose in 2 Gy fractions (EQD2) for commonly used hypofractionated radiation therapy regimens. Low, moderate, and high EQD2 regimens grouped as EQD2 <20 Gy, 20 to 35 Gy, and ≥35 Gy, respectively.

One variable of interest was the RT dose delivered, as several previous studies have demonstrated benefit with administration of higher doses of RT.^17^^,^^26^, ^27^, ^28^, ^29^ For example, 1 retrospective analysis of 655 patients with stage I and II non-Hodgkin lymphoma reported higher in-field control rates for favorable histologies treated with >25 Gy RT (91% vs 78%) and for unfavorable histologies treated with ≥40 Gy (91% vs 61%).^26^ Similarly, a study from MD Anderson Cancer Center reported in-field control rates of 88% versus 71% using ≥40 Gy and <40 Gy RT, respectively.^27^ Still, the more recent retrospective study conducted at Brigham and Women's Hospital/Dana-Farber Cancer Institute failed to detect improved local control with doses >39.6 Gy or twice daily treatments.^13^ The aforementioned BC series did not detect differences in rates of response or local progression for higher RT doses nor single- versus multifractionated RT regimens. Our findings were more similar to the former studies in that higher RT dose (EQD2 ≥35 Gy) was associated with improvements in ORR and TTLF. Given the retrospective nature of this study, it is possible that these findings were influenced by selection bias and the very heterogenous disease characteristics of the cohort. Further analysis is warranted to elucidate specific patient populations that may benefit from higher RT doses. Another particularly interesting finding among patients treated with palliative intent was that short-course RT (4 Gy x 2 fractions) resulted in similar rates of response (Fig. 3). Compared with other palliative RT regimens, TTLF was worse albeit nonstatistically significant. An important caveat, however, is the small number of patients treated with 4 Gy x 2 fractions (n = 14) who were numerically less likely to have bulky disease (17% vs 45% for all other palliative courses; *P* = .11). Ultimately, the desire to achieve an optimal response and local control has to be balanced against transportation and social barriers, poor prognosis, and limited PS. Thus, in patients with poor prognoses and nonbulky disease, 4 Gy x 2 fractions may be appropriate; however, additional study is needed.

Lastly, it is worth noting that a novel role for hypofractionated RT in r/rDLBCL is its use as bridging RT before CART therapy. Patients who are candidates for CART therapy often have progressive symptomatic disease that requires some form of treatment to support them during the variable duration between leukapheresis and CART infusion. Particularly for patients with chemotherapy-refractory disease, bridging RT serves as a practical alternative to systemic treatments. Notably, radiation oncologists are often functioning within a shortened timeframe to plan and deliver RT. The series published by Pinnix et al^30^ reported a median time from leukapheresis to axicabtagene ciloleucel (axi-cel) infusion of 29 days. Additional bridging RT series have demonstrated the use of RT with good tolerance and effect.^31^, ^32^, ^33^ The data presented here further confirm the viability of condensed, hypofractionated courses of bridging RT before CART therapy, and future studies investigating hypofractionated RT in this subpopulation of patients is warranted.

There are several limitations to our study. First, our data were limited to a heterogenous population of patients receiving salvage- or palliative-intent RT for r/rDLBCL at a single institution. The heterogeneity introduces the possibility of selection bias and unmeasured confounding. The multivariable model used in this study likely mitigates, but does not completely control for, confounding variables. Second, more than one-third of patients within the palliative-intent subset expired rapidly and before imaging response assessment, potentially masking the effect of RT (progression of a treated lesion cannot be captured if the patient has expired). Third, the disease outcomes measured herein do not necessarily correlate with symptom relief after RT, and no conclusions should be drawn regarding RT dose or fractionation on palliation of symptoms. Fourth, salvage therapies after RT were not analyzed and could influence disease outcomes in this heterogenous patient cohort. Lastly, the substantial shift in use of hypofractionated RT over the conducted study period reflects an additional source of selection bias associated with hypofractionated treatment regimens. Nevertheless, this study represents all of the patients with r/rDLBLC treated with salvage- or palliative-intent RT over a more than 12-year period at a high-volume tertiary care center and is comparable in size to other studies exploring the utility of RT in this setting.

## Conclusions

In summary, our study demonstrates favorable responses to RT in the palliative and salvage settings, although survival was limited in many patients. Hypofractionated regimens offered comparable ORR, TTLF, and OS to conventional fractionation. Thus, hypofractionation may be particularly useful for patients with a poor prognosis given its abbreviated time courses. As a relatively new RT strategy for this patient population, hypofractionation regimens have yet to be standardized and may benefit from further analysis in larger patient cohorts or prospective clinical trials.

## Appendix Supplementary materials

Image, application 1

## Footnotes

Supplementary material associated with this article can be found in the online version at doi:10.1016/j.adro.2022.101016.
