# Supplementary material for: Radiation Therapy for Relapsed or Refractory Diffuse Large B-Cell Lymphoma: What Is the Right Regimen for Palliation?
Source: Adv Radiat Oncol. 2022 Jul 3;7(6):101016. doi: 10.1016/j.adro.2022.101016 (PMC9677220; doi:10.1016/j.adro.2022.101016)
Supplement: Supplementary file 1 [file mmc1.docx]

**Supplemental Data**

**Supplementary Figure S1.** Most commonly utilized regimens for the A) conventionally fractionated and B) hypofractionated cohorts.

**A**

**B**

Abbreviations: Gy, Gray

**Supplemental Figure S2**. A) TTLF and B) OS as estimated by Kaplan Meier analysis categorized by treatment intent

**Supplemental Figure S3**. A) Acute toxicity per CTCAE v5.0 for conventional fractionation vs hypofractionation. B) Acute toxicity for conventional fractionation vs hypofractionation stratified by EQD2 ≤30 Gy vs > 30 Gy

Abbreviations: Fx, fractionated; Hypofx, hypofractionated

**Supplementary Table S1:** Univariable (UVA) and multivariable (MVA) regression models assessing predictors of overall survival (OS)

| **Variable*** | | **UVA** | | **MVA** | |
| --- | --- | --- | --- | --- | --- |
|  |  | **HR** | **[95% CI, *P*]** | **HR** | **[95% CI, *P*]** |
| **Age at RT** | *<65 years* | reference | |  |  |
| *>65 years* | | 1.26 | [0.87-1.83, 0.22] |  |  |
| **Sex** | *Female* | reference | |  |  |
| *Male* | | 1.28 | [0.88-1.87, 0.19] |  |  |
| **Race** | *White* | reference | |  |  |
| *Black or African American* | | 1.15 | [0.60-2.21, 0.67] |  |  |
| *Other* | | 0.90 | [0.42-1.94, 0.79] |  |  |
| **ECOG PS** | *0-2* | *reference* | | reference | |
| *3-4* | | ***3.06*** | ***[1.95-4.82, <0.0001]*** | **2.27** | **[1.26-4.09, 0.0065]** |
| **Double Hit** | *No* | *reference* | | reference | |
| *Yes* | | ***2.26*** | ***[1.33-3.83, 0.0025]*** | 1.72 | [0.46-6.41, 0.42] |
| **Double Expressor** | *No* | *reference* | | reference | |
| *Yes* | | ***1.69*** | ***[1.06-2.69, 0.028]*** | 1.55 | [0.43-5.53, 0.50] |
| **Cell of Origin** | *ABC* | reference | |  | |
| *GCB* | | 1.22 | [0.81-1.85, 0.35] |  |  |
| **Bulky (>7.5 cm)** | *No* | *reference* | | reference | |
| *Yes* | | ***1.65*** | **[1.10-2.47, 0.015]** | **1.63** | **[1.05-2.55, 0.031]** |
| **Systemic Treatment Lines** | *<2* | reference | |  |  |
| *>2* | | 1.16 | [0.80-1.68, 0.45] |  |  |
| **Intent** | *Salvage* | *reference* | | reference | |
| *Palliative* | | ***2.46*** | ***[1.64-3.71, <0.0001]*** | 1.22 | [0.68-2.19, 0.51] |
| **Fractionation** | *CFX* | *reference* | | reference | |
| *HFX* | | ***2.01*** | ***[1.38-2.92, 0.0003]*** | 1.35 | [0.78-2.32, 0.29] |
| **EQD2** | *<20 Gy* | *reference* | | reference | |
| *20-35 Gy* | | *1.16* | *[0.71-1.91, 0.55]* | 1.69 | [0.89-3.22, 0.11] |
| *>35 Gy* | | ***0.37*** | ***[0.23-0.64, 0.0002]*** | 1.00 | [0.47-2.14, 1.00] |
| **RT Site** | *Head & Neck* | *reference* | | reference | |
| *Thorax* | | *1.86* | *[0.85-4.09, 0.12]* | 2.00 | [0.84-4.79, 0.12] |
| *Abdomen/Pelvis* | | ***2.12*** | ***[1.04-4.33, 0.039]*** | 1.32 | [0.61-2.88, 0.48] |
| *Spine* | | ***2.72*** | ***[1.22-6.06, 0.015]*** | 1.34 | [0.54-3.30, 0.52] |
| *Extremities* | | ***2.48*** | ***[1.03-5.98, 0.044]*** | 2.25 | [0.84-6.00, 0.11] |
| *Multiple Sites* | | *1.53* | *[0.54-4.30, 0.42]* | 1.05 | [0.34-3.25, 0.93] |
| *Other* | | *1.67* | *[0.21-13.20, 0.63]* | 1.04 | [0.12-8.73, 0.97] |
| **RT Technique** | *3DCRT* | *reference* | | reference | |
| *Electron* | | *NE* | *[NE, 0.98]* | NE | [NE, 0.98] |
| *IMRT/VMAT* | | ***0.46*** | ***[0.29-0.73, 0.001]*** | 0.65 | [0.36-1.15, 0.14] |
| *Proton* | | ***0.42*** | ***[0.18-0.98, 0.044]*** | 0.61 | [0.22-1.67, 0.33] |
| *Combined* | | *0.95* | *[0.47-1.91, 0.89]* | 1.22 | [0.50-3.00, 0.66] |
| **RT Completion** | *Yes* | *reference* | | reference | |
| *No* | | ***3.28*** | ***[2.04-5.29, <0.0001]*** | **3.74** | **[2.04-6.84, <0.0001]** |
| **Treatment Location** | *Satellite* | reference | |  |  |
| *Main Site* | | 1.43 | [0.87-2.33, 0.16] |  |  |
| **Treatment Year** | *<2015* | reference | |  |  |
| *>2015* | | 1.02 | [0.70-1.49, 0.91] |  |  |
|  | |  |  |  |  |

Notes: *Cox proportional hazards models were used for OS (158 observations, 93% of total 169 unique patients) with calculated hazard ratios (HRs). Patients with missing values at various covariates were included in models to maintain statistical power but associated HRs for missing values are not shown because not clinically meaningful. *Italicized* variables in UVA were included in MVA as they met pre-determined cut-off *P* <0.10. Abbreviations: OS, overall survival; HR, hazard ratio; CI, confidence interval; UVA, univariable analysis; MVA, multivariable analysis; RT, radiotherapy; ECOG, Eastern Cooperative Oncology Group; PS, performance status; cm, centimeter; CFX, conventional fractionation (<2.5 Gy/fraction); HFX, hypofractionation (>2.5 Gy/fraction); Gy, Gray; EQD2, equivalent dose in 2 Gy fractions; 3DCRT, 3D conformal radiotherapy; IMRT, intensity-modulated radiotherapy; VMAT, volumetric modulated arc therapy; NE, not estimable.

**Supplementary Table S2**. Number of most severe acute grade 1-5 toxicities

| Toxicity | 1 | Toxicity Grade  2 | 3 |
| --- | --- | --- | --- |
| Constitutional | 98 (51) | 38 (20) | 7 (4) |
| HEENT/Respiratory | 27 (14) | 17 (9) | 5 (3) |
| Gastrointestinal | 78 (41) | 22 (12) | 4 (2) |
| Neurologic/Psychiatric | 26 (14) | 11 (6) | 0 (0) |
| Dermatologic | 41 (21) | 16 (8) | 0 (0) |
| Other | 17 (9) | 10 (5) | 3 (2) |

N=191 RT courses. 14 patients did not have toxicity data available.
